# Occupational Exposure to Carbofuran and the Incidence of Cancer in the Agricultural Health Study

**DOI:** 10.1289/ehp.7451

**Published:** 2004-12-02

**Authors:** Matthew R. Bonner, Won Jin Lee, Dale P. Sandler, Jane A. Hoppin, Mustafa Dosemeci, Michael C. R. Alavanja

**Affiliations:** ^1^Occupational and Environmental Epidemiology Branch, Division of Cancer Epidemiology and Genetics, National Cancer Institute, Bethesda, Maryland, USA; ^2^Department of Preventive Medicine, College of Medicine, Korea University, Seoul, Korea; ^3^Epidemiology Branch, National Institute of Environmental Health Sciences, Research Triangle Park, North Carolina, USA

**Keywords:** agriculture, cancer incidence, carbofuran, lung cancer, pesticides

## Abstract

Carbofuran is a carbamate insecticide registered for use on a variety of food crops including corn, alfalfa, rice, and tobacco. An estimated 5 million pounds of carbofuran is used annually in the United States, and 45% of urban African-American women have detectable levels of carbofuran in their plasma. Nitrosated carbofuran has demonstrated mutagenic properties. We examined exposure to carbofuran and several tumor sites among 49,877 licensed pesticide applicators from Iowa and North Carolina enrolled in the Agricultural Health Study. We obtained information regarding years of use, frequency of use in an average year, and when use began for 22 pesticides using self-administered questionnaires. Poisson regression was used to calculate rate ratios (RR) and 95% confidence intervals (CIs) adjusting for potential confounders. Lung cancer risk was 3-fold higher for those with > 109 days of lifetime exposure to carbofuran (RR = 3.05; 95% CI, 0.94–9.87) compared with those with < 9 lifetime exposure days, with a significant dose–response trend for both days of use per year and total years of use. However, carbofuran use was not associated with lung cancer risk when nonexposed persons were used as the referent. In addition, carbofuran exposure was not associated with any other cancer site examined. Although carbamate pesticides are suspected human carcinogens, these results should be interpreted cautiously because there was no *a priori* hypothesis specifically linking carbofuran to lung cancer.

Carbofuran (2,3-dihydro-2,2-dimethylbenzofuran-7-yl methylcarbamate) is a carbamate insecticide registered for use on a variety of food crops including corn, alfalfa, rice, and tobacco (Tobin 1970). An estimated 5 million pounds of carbofuran are used annually in the United States, 48% of which is used on corn crops (Thelin and Gianessi 2000). In addition to agriculturally related exposure, the general U.S. population may also be commonly exposed to carbofuran. Forty-five percent of urban African-American women and their newborns had detectable levels of carbofuran in maternal plasma and umbilical cord blood (Whyatt et al. 2003).

Carbofuran has been demonstrated to have weak mutagenic activity in some, but not all, strains of *Salmonella typhimurium* (Hour et al. 1998; Moriya et al. 1983). Carbofuran induces chromosomal aberrations and micronucleus formation in exposed mice (Chauhan et al. 2000) and *N*-nitrosocarbofuran, derived from the nitrosation of carbofuran, has demonstrated mutagenic properties (Yoon et al. 2001). The evidence from animal models is inconclusive. Two studies demonstrated that carbofuran was able to induce lymphoma in Swiss mice (Borzsonyi and Pinter 1977; Borzsonyi et al. 1976), but carcinogenicity of carbofuran was not evident in several 2-year dietary studies conducted on rats (Gupta 1994).

In addition to the potential carcinogenicity of *N*-nitrosocarbofuran, carbamate pesticides have been shown to impair immunity in mice (Barnett et al. 1980; Street and Sharma 1975) and in humans (Fiore et al. 1986). Several epidemiologic investigations have examined exposure to carbamate pesticides, including carbofuran, and the risk of cancer. McDuffie et al. (2001) found elevated risk for non-Hodgkin lymphoma (NHL) associated with the use of carbamate pesticides [odds ratio (OR) = 1.9; 95% confidence interval (CI), 1.2–3.0], but not with carbofuran specifically (OR = 1.6; 95% CI, 0.7–3.9). Zheng et al. (2001) observed elevated risk among farmers who used carbofuran (OR = 1.6; 95% CI, 1.1–2.3). In a nested case–control study of structural pest control workers in Florida, Pesatori et al. (1994) reported an increase in the OR for lung cancer among those who used carbamate insecticides (OR = 16.3; 95% CI, 2.2–122.5). Increased risk of lung cancer was not evident, however, in a population-based case–control study among residents of Saskatchewan, Canada (McDuffie et al. 1990). Considering the limited epidemiologic data on carbofuran and cancer, we examined the relationship between occupational exposure to carbofuran and several tumor sites in the Agricultural Health Study.

## Materials and Methods

A detailed description the Agricultural Health Study (AHS) has been previously published (Alavanja et al. 1996). Briefly, the AHS is a prospective cohort study of 57,311 licensed restricted-use pesticide applicators and 32,347 of their spouses in Iowa and North Carolina. Licensed pesticide applicators include private applicators who are farmers and commercial applicators who are employed by pest control companies or businesses that use pesticides such as warehouse operators and grain millers. Recruitment started in December 1993 and ended 4 years later in December 1997. The National Death Index and state death registries were used to ascertain the vital status of cohort members. Incident cancers diagnosed between December 1993 and 31 December 2001 were identified through tumor registries and coded using the *International Classification of Diseases for Oncology* (ICD-O-2) (Percy et al. 1990). The average follow-up time was 6.4 years. Participants (*n* = 946) who moved out of either Iowa or North Carolina were censored in the year they moved. All participants provided informed consent; the protocol was approved by all appropriate Institutional Review Boards.

### Exposure assessment.

Study participants were asked to complete a self-administered questionnaire at the time of enrollment. We obtained information on 50 pesticides. Detailed data about years of use, frequency of use in an average year, and when use began were collected for 22 pesticides including carbofuran, and information on ever/never use of 28 other pesticides was collected at the time of enrollment. In addition, information regarding application methods and the use of personal protective equipment was collected. Participants also supplied information about important potential confounders such as smoking habits, alcohol intake, fruit and vegetable consumption, other agricultural activities, and non-farm–related occupational exposures. A previous analysis of the reliability of the AHS questionnaire showed that the level of agreement for pesticide use was similar to other factors routinely estimated with epidemiologic questionnaires (Blair et al. 2002).

For these analyses, we estimated exposure with total lifetime exposure-days to carbofuran. Lifetime exposure-days was defined as the product of the number of years a participant personally mixed or applied carbofuran and the number of days in an average year that carbofuran was used. In addition, we incorporated an algorithm developed by Dosemeci et al. (2002) to estimate an exposure intensity score and applied it to lifetime exposure-days metric. Briefly, the intensity score was designed to incorporate aspects of pesticide use that can modify actual exposure, including whether an applicator personally mixed or prepared the pesticides for application, what type of application methods were used, the repair of pesticide application equipment, and the use of personal protective equipment during these activities. Dermal absorption is generally considered the major route of exposure for pesticide applicators (Maroni et al. 2000; Tobin 1970). Therefore, the intensity score heavily weighted the use of protective gloves and to a lesser extent protective clothing. We calculated intensity-weighted exposure-days as the product of the intensity score and total lifetime exposure-days. In addition to these exposure metrics, we also assessed the frequency (i.e., number of days/year applied) and the duration (total number of years applied) of carbofuran exposure in relation to cancer risk.

### Statistical analysis.

Prevalent cancer cases (*n* = 1,074) and applicators who failed to provide information about carbofuran use (*n* = 6,360) were excluded, leaving 49,877 cohort members from this analysis. Most of the subjects who were missing information on carbofuran use and other potential confounders were from North Carolina (64%). Two reference groups were used for these analyses: pesticide applicators who reported never using carbofuran and pesticide applicators whose use of carbofuran was in the lowest tertile of exposure.

We used Poisson regression to calculate rate ratios (RR) and 95% CIs. Lifetime exposure-days and intensity-weighted lifetime exposure-days to carbofuran were categorized into tertiles based on the distribution in all the cancer cases. The highest tertile was then divided at its midpoint to increase the resolution at higher exposure levels. We limited analyses to tumor sites where there were more than five cases in each category of exposure. Models were adjusted for age at enrollment (< 40, 40–49, 50–59, ≥ 60 years), sex, education (≤ high school graduate, > high school graduate), smoking (by pack-years: never, ≤ 14, > 14), alcohol consumption during the last 12 months (yes/no), family history of cancer (yes/no), year at enrollment, state of residence (Iowa/North Carolina), and the five pesticides most highly correlated with carbofuran use [permethrin (crop), *S*-ethyl dipropylthiocarbamate (EPTC), chlorpyrifos, fonofos, and trichlorfon: never, low, high exposure]. The correlation coefficients for these five pesticides ranged between 0.69 (permethrin) and 0.85 (trichlorfon). The cut point that dichotomized low and high exposure for each pesticide correlated with carbofuran was determined by the median for lifetime exposure-days for that particular pesticide. We based the cut points for days of use per year and years of use on the categorical responses to the following questions: “In an average year when you personally used this pesticide, how many days did you use it?” (< 5 days; 5–9 days; 10–19 days; 20–39 days; 40–59 days; 60–150 days; or > 150 days) and “How many years did you personally mix or apply this pesticide?” (≤ 1 year or less; 2–5 years; 6–10 years; 11–20 years; 21–30 years; or > 30 years). For the analysis, we collapsed the upper categories to ensure that there were approximately five or more cases in each category.

We determined the most parsimonious model (reduced) with −2 log-likelihood ratio tests by removing each covariate from the saturated (full) model and retaining only those variables that resulted in significant −2 log-likelihood ratio (Hosmer and Lemeshow 1989). The most parsimonious model included age, smoking (never, < 14 pack-years, and ≥ 14 pack-years of smoking), family history of cancer, and trichlorofon and permethrin exposure.

To further control for potential confounding by smoking, we also adjusted for several other smoking variables including smoking status (never, former, current), pack-years of smoking, duration of smoking, and number of cigarettes smoked per day. The inclusion of these additional smoking variables did not appreciably alter the risk estimates and were not retained in the models. Linear trends were assessed using the *p*-value of the coefficient of the exposure treated as a continuous value using the median value for each tertile of exposure in the models also adjusting for covariates (Breslow and Day 1987). Tests for interaction were performed by determining the significance of the coefficient of the product term of the exposure and the purported effect modifier.

## Results

Twenty-five percent of the pesticide applicators reported ever using carbofuran. Demographic characteristics of the non-carbofuran exposed and carbofuran exposed [categorized as low (tertile 1) and high exposure (tertiles 2 and 3)] are depicted in Table 1. The non-carbofuran exposed tended to be younger than either the low- or high-exposed carbofuran cohorts. The nonexposed were also more likely to be female than the exposed, although there were few women applicators in the study overall. Smoking status (never, former, or current), alcohol consumption in the last 12 months, attained education, state of residence, years of follow-up, and family history of cancer were all similar between the three groups. The mean number of smoking pack-years; however, sequentially increased between nonexposed, low-exposed, and the high-exposed groups. Cohort members exposed to carbofuran were more likely than nonexposed cohort members to be involved in corn production. Additionally, those exposed to carbofuran used more types of pesticides than non-carbofuran–exposed subjects.

We report on all cancer sites combined and tumor sites where sufficient numbers (at least five cases per cell) of cases occurred during follow-up to warrant statistical analyses: all lymphatic–hematopoietic cancers (Hodgkin, non-Hodgkin, multiple myeloma, and leukemia), NHL, and colon, lung, and prostate cancers.

Carbofuran exposure was not associated with the incidence of all cancers combined (Table 2) or with any tumor site examined except lung cancer. Lung cancer risk appeared to be positively associated with exposure to carbofuran when the low exposed were used as the reference group, although a test of the linear trend was not significant (*p* for trend = 0.07). The lung cancer rate ratio was increased 3-fold among those with more than 109 lifetime-days of use (RR = 3.05; 95% CI, 0.94–9.87). When the nonexposed were used as the reference group, however, exposure to carbofuran was not associated with the lung cancer rate ratio.

An exposure–response relationship with the intensity-weighted lifetime exposure-days was not clearly evident for lung cancer (Table 3). Although the upper category of the intensity-weighted lifetime exposure-days suggests an increase in the relative risk, the exposure–response gradient was not monotonic. Regarding the other cancer sites examined, there was no evidence of an association with intensity-weighted lifetime exposure-days when either the nonexposed or the low-exposed subjects were used as the referent (data not shown).

The risk of lung cancer also increased when the frequency of exposure (number of days of carbofuran use/year) and duration of exposure (number of years carbofuran was used) were examined separately (Table 3). However, the risk was only elevated in applicators who used carbofuran for > 10 years and for > 10 applications days per year.

To further examine and characterize the association between carbofuran exposure and lung cancer, we stratified by smoking status (never, former, and current), state of residence (Iowa and North Carolina), histology (adenocarcinoma and non-adenocarcinoma), and applicator type (farmer and commercial). The analyses stratified by smoking status were limited in that only one case of lung cancer was identified among never smokers and precluded an analysis restricted to never smokers. The risk estimates increased as exposure increased for both former and current smokers (Table 4), and the *p* for interaction was not significant (*p* = 0.36). Carbofuran exposure was associated with nonsignificant increases in risk in both Iowa (2nd tertile: RR = 3.79, 95% CI, 0.73–19.55; 3rd tertile: RR = 5.90, 95% CI, 1.25–27.81) and North Carolina (2nd tertile: RR = 0.91, 95% CI, 0.20–4.05; 3rd tertile: RR = 2.49, 95% CI, 0.77–8.14). Although the point estimates of risk were greater in Iowa, the *p*-value for the interaction between state and carbofuran exposure was not significant (*p* for interaction = 0.53). Rate ratios were increased for both adenocarcinoma (2nd tertile: RR = 3.95, 95% CI, 0.41–38.02; 3rd tertile: RR = 7.87, 95% CI, 0.94–65.62) and non-adenocarcinoma (2nd tertile: RR = 1.35, 95% CI, 0.39–4.68; 3rd tertile: RR = 2.90, 95% CI, 1.0–8.36). Although the risk was considerably higher for adenocarcinoma, the *p* for interaction between histology and carbofuran use was not significant (*p* = 0.32). There was no evidence that applicator type either confounded or modified the association between carbofuran and lung cancer risk, although the number of commercial applicator lung cancer cases was low.

## Discussion

An association between carbofuran and lung cancer has not been previously reported. Several studies, however, have found pesticides (Brownson et al. 1993; Wesseling et al. 1999) and more specifically carbamate pesticides (Pesatori et al. 1994) to be associated with lung cancer, although not all studies have reported this association (McDuffie et al. 1990). In our study, lung cancer was associated with lifetime exposure-days where risk increased across exposure categories to more than a 3-fold increase in the RR in the highest category when compared with those who had applied < 9 lifetime exposure-days. The risk estimates were also elevated when the components of the lifetime exposure-days exposure metric were considered separately. Lung cancer risk, however, was not associated with carbofuran exposure when the intensity-weighted exposure-days metric was used or when non-carbofuran–exposed pesticide applicators were used as the referent.

This inconsistency between the lung cancer risk estimates when nonexposed subjects were used as the referent may be caused partly by differences between nonexposed and low-exposed groups with regard to unknown factors. Initial descriptive analyses indicated that the nonexposed and the low-exposed groups had substantial differences with regard to corn production and the total number of pesticides used. The observed differences between those with carbofuran exposure and those without carbofuran exposure raise the possibility of confounding due to other unmeasured differences between the groups. Given these differences, the low-exposed subjects may be a more appropriate reference group, although the low-exposed group may be biased as well. In addition, the inconsistency observed between the lifetime exposure-days and intensity-weighted lifetime exposure-days metrics may have occurred because the AHS intensity-weighted algorithm greatly weights dermal exposure, and this route may be less appropriate for sites where the respiratory tract is the predominant exposure route, such as the lung. Further, the intensity-weighted algorithm, as constructed, also reflects more recent use of personal protective equipment and application methods. Malignant neoplasms generally have a long latency period. To the extent that recent exposure intensity does not accurately reflect past activities, the algorithm may increase exposure misclassification rather than reduce it.

The association between lung cancer and carbofuran exposure that we observed when the low-exposed group was used as the referent is unlikely to be confounded by smoking because pack-years of smoking was not correlated with lifetime exposure-days (*r* = 0.03) or intensity-weighted lifetime exposure-days (*r* = 0.02). Furthermore, we adjusted for smoking (never, < 14 pack-years, and ≥ 14 pack-years) in the models. Even when we used pack-years as a continuous variable or a combination of smoking status (never, former, current), number of cigarettes smoked per day and number of years smoked, the risk estimates were similar with each respective model. We also stratified by smoking status and found that the association was relatively consistent between former and current smokers. There were too few lung cancer cases to determine whether carbofuran was associated with lung cancer independent of smoking. Therefore, we cannot rule out the possibility that the association between carbofuran and the risk of lung cancer is limited to smokers and former smokers.

Agricultural exposure to endotoxin from rearing livestock has been hypothesized to reduce the risk of lung cancer (Lange et al. 2003a, 2003b, 2003c; Mastrangelo et al. 1996). Although we did not formally assess exposure to endotoxin, we conducted an analysis stratifying the cohort into those who were engaged in animal husbandry and those who were not. There was no indication that animal husbandry modified the effect of carbofuran use on lung cancer risk. In addition, engaging in animal husbandry did not confound the association between carbofuran use and lung cancer because the RRs were not altered when we included a binary animal husbandry variable in the model.

Several previous investigations of NHL have observed increases in risk associated with carbofuran exposure (McDuffie et al 2001; Zheng et al. 2001). In addition, results from several animal models support the hypothesis that exposure to carbofuran could be a risk factor for NHL (Borzsonyi and Pinter 1977; Borzsonyi et al. 1976). We found little evidence to support an association between NHL and carbofuran exposure, although relatively few cases of NHL had accrued at the time of this analysis.

There is evidence that carcinogenic *N*-nitrosocarbofuran is formed from carbofuran and nitrites in the stomach. *A priori*, we expected carbofuran exposure to be associated with increased risk for stomach cancer; however, at the time these analyses were conducted, too few cases of stomach cancer had occurred in the carbofuran exposed cohorts for meaningful analysis.

There are some important limitations of this study. Although the incidence of cancers will increase as the cohort ages, currently we remain constrained by small numbers of cases for many tumor sites. For instance, only five cases of stomach cancer with exposure to carbofuran were available for analysis. The resulting statistical imprecision makes interpretation of risk estimates difficult in some instances. Another potential concern in prospective studies is loss to follow-up. However, losses to follow-up (< 2%) were few and were unlikely to substantially bias the risk estimates. In addition, pesticides are commonly used as formulations where only a percentage of the total product applied is the active ingredient. Given that pesticides are applied as complex mixtures or solutions, we cannot rule out the possibility that the combination or the “inert” ingredients are the actual carcinogenic compound(s).

The strengths of this study include the prospective design, where exposure to pesticides was determined before the onset of disease, thereby eliminating the potential for recall bias. In addition, the exposure metrics used in this study represent a major improvement in the classification of pesticide exposure over previous studies, although, undoubtedly, some exposure misclassification is present in our estimates as well.

Multicolinearity between pesticides used may be another potential limitation of this study. We assessed exposure to 50 pesticides in registered pesticide applicators who, on average, used numerous pesticides. Because it is possible that carbofuran use is related to several other pesticides, we identified the five most correlated pesticides and adjusted for them in the model. Overall, exposure to other individual pesticides was highly correlated with carbofuran exposure. The correlation coefficients ranged between 0.69 (permethrin) and 0.85 (trichlorfon). However, these pesticides did not confound the association between carbofuran and lung cancer because the risk estimates were not altered when they were removed from the model. In addition, we also adjusted for cumulative lifetime application days of all pesticides, which did not appreciably alter the risk estimates.

Overall, we examined the risk of several cancer sites in relation to the carbofuran exposure. Carbofuran is a carbamate insecticide with questionable carcinogenic properties in animals. The parent compound does not seem to be genotoxic. However, the metabolites of carbofuran may be mutagenic, and there is good evidence that nitrosated carbofuran is mutagenic. This study suggests that carbofuran may be associated with an increase in the incidence of lung cancer. Conversely, carbofuran exposure was not associated with other tumor sites investigated. The results for lung cancer are provocative but should be interpreted cautiously in light of the paucity of other studies to corroborate these findings, and a reevaluation of carbofuran in the AHS cohort once more cancer cases have accrued is warranted.

## Figures and Tables

**Table 1:** Selected characteristics of applicators, by carbofuran exposure [no. (%)] in the AHS (1993–1997).

Characteristic	Nonexposed	Low exposed	High exposed
Age (years)			
< 40	14,023 (37.6)	1,032 (21.8)	1,739 (22.1)
40–49	10,217 (27.4)	1,470 (31.0)	2,532 (32.1)
50–59	6,802 (18.3)	1,216 (25.7)	2,056 (26.1)
≥ 60	6,210 (16.7)	1,016 (21.5)	1,557 (19.7)
Sex			
Male	36,069 (96.8)	4,698 (99.2)	7,819 (99.2)
Female	1,190 (3.2)	36 (0.8)	65 (0.8)
State			
Iowa	25,459 (68.3)	3,421 (72.3)	4,908 (62.3)
North Carolina	11,800 (31.7)	1,313 (27.7)	2,976 (37.7)
Applicator type			
Farmer	33,341 (89.5)	4,574 (96.6)	7,355 (93.3)
Commercial	3,918 (10.5)	160 (3.4)	529 (6.7)
Smoking			
Never	19,976 (54.0)	2,509 (53.2)	4,056 (51.6)
Former	10,587 (28.7)	1,577 (33.4)	2,560 (32.6)
Current	6,396 (17.3)	635 (13.5)	1,241 (15.8)
Alcohol use*^a^*			
Yes	25,352 (69.0)	3,260 (69.1)	5,290 (67.8)
Education			
≤ High school	21,270 (57.2)	2,503 (53.0)	4,372 (55.5)
> High school	15,897 (42.8)	2,219 (47.0)	3,504 (44.5)
Family history of cancer			
Yes	13,339 (38.0)	2,099 (46.5)	3,404 (45.9)
Corn production			
Yes	24,967 (67.0)	3,801 (80.0)	6,226 (79.0)
Other pesticide use			
Trichlorofon	305 (0.8)	37 (0.8)	160 (2.1)
Fonofos	5,410 (14.5)	1,591 (34.4)	2,969 (38.6)
Chlorpyrifos	12,908 (34.7)	2,534 (53.8)	4,982 (63.5)
EPTC	6,112 (16.8)	1,351 (29.5)	2,417 (31.8)
Permethrin*^b^*	4,078 (11.1)	913 (19.9)	2,060 (27.1)
Person-years	240549.2	29867.9	50852.2
No. of other pesticides used*^c^*	11.5 ± 6.6	18.3 ± 6.6	20.4 ± 7.2
Follow-up (years)*^c^*	6.5 ± 1.4	6.3 ± 1.4	6.5 ± 1.4
Smoking (pack-years)*^c^*			
Former smokers	15.4 ± 20.1	15.0 ± 18.9	15.8 ± 20.2
Current smokers	22.0 ± 19.9	24.9 ± 21.3	27.0 ± 22.2

a Reported alcohol consumption within the last 12 months.

b Permethrin for use on crops.

c Mean ± SD.

**Table 2:** RRs for selected cancers, by lifetime exposure-days to carbofuran among AHS (1993–1997) applicators with nonexposed and low-exposed groups as referents.

Lifetime exposure days*^a^*	Cases (*n*)	Nonexposed referent RR (95% CI)	Low-exposed referent RR (95% CI)
All cancers			
0	1,012	1.0	
> 0–9	151	0.95 (0.80–1.14)	1.0
10–39	115	0.95 (0.78–1.15)	1.00 (0.78–1.27)
40–109	80	1.05 (0.83–1.33)	1.11 (0.83–1.49)
> 109	51	0.94 (0.70–1.26)	0.96 (0.67–1.37)
Trend*^b^*		0.79	0.94
Lymphatic–hematopoietic cancers			
0	103	1.0	
> 0–9	11	0.68 (0.36–1.30)	1.0
10–39	10	0.82 (0.42–1.60)	1.05 (0.44–2.51)
40–109	11	1.38 (0.72–2.65)	1.56 (0.62–3.92)
> 109	5	0.86 (0.34–2.23)	0.77 (0.23–2.57)
Trend*^b^*		0.93	0.74
Non-Hodgkin lymphoma			
0	44	1.0	
> 0–9	6	0.77 (0.31–1.86)	1.0
10–39	7	1.27 (0.55–2.91)	1.33 (0.44–4.02)
40–109	7	1.40 (0.59–3.30)	1.08 (0.31–3.74)
Trend*^b^*		0.40	0.94
Colon			
0	80	1.0	
> 0–9	10	0.88 (0.45–1.72)	1.0
10–39	9	0.99 (0.49–2.02)	1.03 (0.41–2.56)
40–109	5	0.84 (0.33–2.12)	0.77 (0.25–2.42)
> 109	6	1.34 (0.54–3.31)	1.16 (0.36–3.71)
Trend*^b^*		0.68	0.85
Lung			
0	98	1.0	
> 0–9	6	0.42 (0.18–0.97)	1.0
10–39	8	0.68 (0.33–1.43)	1.61 (0.55–4.69)
40–109	9	1.09 (0.54–2.22)	2.54 (0.85–7.67)
> 109	8	1.38 (0.63–2.99)	3.05 (0.94–9.87)
Trend*^b^*		0.46	0.07
Prostate			
0	372	1.0	
> 0–9	85	1.30 (1.01–1.66)	1.0
10–39	48	0.99 (0.73–1.35)	0.79 (0.55–1.13)
40–109	29	1.03 (0.70–1.53)	0.86 (0.55–1.36)
> 109	17	0.88 (0.53–1.47)	0.73 (0.41–1.31)
Trend*^b^*		0.70	0.34

Rate ratios adjusted for age, sex, education, family history of cancer, smoking, alcohol, year of enrollment, state of residence, and exposure to EPTC, fonofos, trichlorofon, chlorpyrifos, and permethrin.

a Years of use × days of use per year.

b *p*-Value for trend test.

**Table 3:** RRs for lung cancer by carbofuran intensity-weighted lifetime exposure days, exposure frequency (days per year), and exposure duration (years of use) in the AHS (1993–1997).

	Cases (*n*)	Full model RR (95% CI)*^a^*	Reduced model RR (95% CI)*^b^*
Intensity-weighted lifetime exposure days*^c^*			
> 0–63	6	1.0	1.0
64–196	11	2.11 (0.77–5.78)	2.42 (0.89–6.54)
197–487	5	1.19 (0.35–4.03)	1.58 (0.48–5.19)
> 487	9	2.10 (0.69–6.39)	3.40 (1.21–9.58)
Trend*^d^*		0.40	0.23
Days of use/year			
< 5	9	1.0	1.0
5–9	9	1.53 (0.59–3.95)	1.67 (0.66–4.21)
10–19	9	2.98 (1.07–8.33)	3.84 (1.52–9.71)
≥ 20	4	4.13 (1.13–15.08)	5.63 (1.73–18.35)
Trend*^d^*		< 0.01	< 0.01
Years of use			
≤ 5	16	1.0	1.0
6–10	6	0.80 (0.30–2.10)	1.00 (0.39–2.55)
> 10	9	1.95 (0.80–4.77)	3.00 (1.32–6.81)
Trend*^d^*		0.02	< 0.01

a Rate ratios adjusted for age, sex, education, family history of cancer, smoking, alcohol, year of enrollment, state of residence, and exposure to EPTC, fonofos, trichlorofon, chlorpyrifos, and permethrin.

b Rate ratios adjusted for age, smoking (never, < 14 pack-years, ≥ 14 pack-years), family history of cancer, and exposure to trichlorofon and permethrin.

c Years of use × days of use per year × intensity score.

d *p*-Value for trend test.

**Table 4:** RRs for lung cancer and lifetime exposure-days to carbofuran, by smoking status in the AHS (1993–1997).

	Former smokers		Current smokers	
Lifetime exposure days*^a^*	Cases (*n*)	RR (95% CI)	Cases (*n*)	RR (95% CI)
> 0–9	3	1.0	3	1.0
10–39	3	1.87 (0.42–8.37)	4	1.75 (0.39–7.90)
> 39	11	4.88 (1.36–17.52)	6	2.49 (0.62–10.00)
Trend*^b^*		< 0.01		0.23
*p* for interaction	0.36			

Rate ratios adjusted for age, smoking (never, < 14 pack-years, ≥ 14 pack-years), family history of cancer, exposure to trichlorofon and permethrin.

a Years of use × days of use per year.

b *p*-Value for trend test.

## References

[b1-ehp0113-000285] Alavanja MC, Sandler DP, McMaster SB, Zahm SH, McDonnell CJ, Lynch CF (1996). The Agricultural Health Study. Environ Health Perspect.

[b2-ehp0113-000285] Barnett JB, Spyker-Cranmer JM, Avery DL, Hoberman AM (1980). Immunocompetence over the lifespan of mice exposed *in utero* to carbofuran or diazinon. I. Changes in serum immunoglobulin concentrations. J Environ Pathol Toxicol.

[b3-ehp0113-000285] Blair A, Tarone R, Sandler D, Lynch CF, Rowland A, Wintersteen W (2002). Reliability of reporting on lifestyle and agricultural factors by a sample of participants in the Agricultural Health Study from Iowa. Epidemiology.

[b4-ehp0113-000285] Borzsonyi M, Pinter A (1977). The carcinogenicity of *N*-nitroso compounds formed endogenously in mice from benzimidazole carbamate pesticides. Neoplasma.

[b5-ehp0113-000285] Borzsonyi M, Pinter A, Surjan A, Farkas I (1976). Transplacental induction of lymphomas in Swiss mice by carbendazim and sodium nitrite. Int J Cancer.

[b6-ehp0113-000285] Breslow NE Day NE 1987. Statistical Methods in Cancer Research II–The Design and Analysis of Cohort Studies. Lyon:International Agency for Research on Cancer. 3329634

[b7-ehp0113-000285] Brownson RC, Alavanja MC, Chang JC (1993). Occupational risk factors for lung cancer among nonsmoking women: a case-control study in Missouri (United States). Cancer Causes Control.

[b8-ehp0113-000285] Chauhan LK, Pant N, Gupta SK, Srivastava SP (2000). Induction of chromosome aberrations, micronucleus formation and sperm abnormalities in mouse following carbofuran exposure. Mutat Res.

[b9-ehp0113-000285] Dosemeci M, Alavanja MC, Rowland AS, Mage D, Zahm SH, Rothman N (2002). A quantitative approach for estimating exposure to pesticides in the Agricultural Health Study. Ann Occup Hyg.

[b10-ehp0113-000285] Fiore MC, Anderson HA, Hong R, Golubjatnikov R, Seiser JE, Nordstrom D (1986). Chronic exposure to aldicarb-contaminated groundwater and human immune function. Environ Res.

[b11-ehp0113-000285] Gupta RC (1994). Carbofuran toxicity. J Toxicol Environ Health.

[b12-ehp0113-000285] Hosmer DW Lemeshow S 1989. Applied Logistic Regression. New York:John Wiley & Sons.

[b13-ehp0113-000285] Hour TC, Chen L, Lin JK (1998). Comparative investigation on the mutagenicities of organophosphate, phthalimide, pyrethroid and carbamate insecticides by the Ames and lactam tests. Mutagenesis.

[b14-ehp0113-000285] Lange JH, Mastrangelo G, Fedeli U, Fadda E, Rylander R, Lee E (2003a). Endotoxin exposure and lung cancer mortality by type of farming: is there a hidden dose-response relationship?. Ann Agric Environ Med.

[b15-ehp0113-000285] Lange JH, Mastrangelo G, Fedeli U, Rylander R, Christiani DC (2003b). There is an alternative reason for lower-than-expected rates of lung cancer in farmers. Arch Environ Health.

[b16-ehp0113-000285] Lange JH, Rylander R, Fedeli U, Mastrangelo G (2003c). Extension of the “hygiene hypothesis” to the association of occupational endotoxin exposure with lower lung cancer risk. J Allergy Clin Immunol.

[b17-ehp0113-000285] Maroni M, Colosio C, Ferioli A, Fait A (2000). Biological monitoring of pesticide exposure: a review. Introduction. Toxicology.

[b18-ehp0113-000285] Mastrangelo G, Marzia V, Marcer G (1996). Reduced lung cancer mortality in dairy farmers: is endotoxin exposure the key factor?. Am J Ind Med.

[b19-ehp0113-000285] McDuffie HH, Klaassen DJ, Dosman JA (1990). Is pesticide use related to the risk of primary lung cancer in Saskatchewan?. J Occup Med.

[b20-ehp0113-000285] McDuffie HH, Pahwa P, McLaughlin JR, Spinelli JJ, Fincham S, Dosman JA (2001). Non-Hodgkin’s lymphoma and specific pesticide exposures in men: cross-Canada study of pesticides and health. Cancer Epidemiol Biomarkers Prev.

[b21-ehp0113-000285] Moriya M, Ohta T, Watanabe K, Miyazawa T, Kato K, Shirasu Y (1983). Further mutagenicity studies on pesticides in bacterial reversion assay systems. Mutat Res.

[b22-ehp0113-000285] Percy C Van Holten V Muir C 1990. International Classification of Diseases for Oncology. Geneva:World Health Organization.

[b23-ehp0113-000285] Pesatori AC, Sontag JM, Lubin JH, Consonni D, Blair A (1994). Cohort mortality and nested case-control study of lung cancer among structural pest control workers in Florida (United States). Cancer Causes Control.

[b24-ehp0113-000285] Street JC, Sharma RP (1975). Alteration of induced cellular and humoral immune responses by pesticides and chemicals of environmental concern: quantitative studies of immunosuppression by DDT, aroclor 1254, carbaryl, carbofuran, and methylparathion. Toxicol Appl Pharmacol.

[b25-ehp0113-000285] Thelin GP Gianessi LP 2000. Method for estimating pesticide use for county areas of the conterminous United States. Sacramento, CA:U.S. Geological Survey.

[b26-ehp0113-000285] Tobin JS (1970). Carbofuran: a new carbamate insecticide. J Occup Med.

[b27-ehp0113-000285] Wesseling C, Antich D, Hogstedt C, Rodriguez AC, Ahlbom A (1999). Geographical differences of cancer incidence in Costa Rica in relation to environmental and occupational pesticide exposure. Int J Epidemiol.

[b28-ehp0113-000285] Whyatt RM, Barr DB, Camann DE, Kinney PL, Barr JR, Andrews HF (2003). Contemporary-use pesticides in personal air samples during pregnancy and blood samples at delivery among urban minority mothers and newborns. Environ Health Perspect.

[b29-ehp0113-000285] Yoon JY, Oh SH, Yoo SM, Lee SJ, Lee HS, Choi SJ (2001). *N*-nitrosocarbofuran, but not carbofuran, induces apoptosis and cell cycle arrest in CHL cells. Toxicology.

[b30-ehp0113-000285] Zheng T, Zahm SH, Cantor KP, Weisenburger DD, Zhang Y, Blair A (2001). Agricultural exposure to carbamate pesticides and risk of non-Hodgkin lymphoma. J Occup Environ Med.

